# LncRNA *H19* gene rs2839698 polymorphism is associated with a decreased risk of colorectal cancer in a Chinese Han population: A case‐control study

**DOI:** 10.1002/jcla.23311

**Published:** 2020-03-24

**Authors:** Bingqu Yu, Jiayuan Chen, Chenfeng Hou, Lei Zhang, Jie Jia

**Affiliations:** ^1^ Department of Gastroenterology Wenzhou Hospital of Integrated Traditional Chinese and Western Medicine Wenzhou China; ^2^ Department anorectal surgery The Second Affiliated Hospital of Zhejiang Chinese Medical University Hangzhou China

**Keywords:** case‐control study, colorectal cancer, lncRNA H19, rs2839698 polymorphism

## Abstract

**Background:**

Long non‐coding RNA (lncRNA) H19 is involved in the carcinogenesis, progression, and metastasis of colorectal cancer (CRC). Recently, a few studies explored the relationship between lncRNA *H19* gene rs2839698 polymorphism and CRC risk, but with conflicting findings.

**Materials and methods:**

A case‐control study with 315 CRC cases and 441 controls was designed in a Chinese population. Genotyping was performed using PCR‐RFLP.

**Results:**

It was found rs2839698 polymorphism was associated with a decreased risk of CRC (AA vs GG: OR, 0.73; 95% CI, 0.54‐0.98; *P* = .037; A vs G: OR, 0.78; 95% CI, 0.63‐0.96; *P* = .021). Stratified analyses indicated this positive association was also significant in the non‐smokers (AA vs GG: OR, 0.49; 95% CI, 0.25‐0.93; *P = *.029), non‐drinkers, those aged ≥ 60 years, and overweight individuals (BMI ≥ 24). In addition, rs2839698 polymorphism was also related to the lymph node metastasis (AA vs GG: OR, 0.43; 95% CI, 0.21‐0.88; *P = *.019) and tumor size (AA vs GG: OR, 0.42; 95% CI, 0.20‐0.88; *P = *.020) for patients with CRC.

**Conclusion:**

To sum up, the lncRNA *H19* gene rs2839698 polymorphism decreases the risk of CRC in Chinese individuals, especially among the non‐smokers, non‐drinkers, individuals aged ≥ 60 years, and overweight individuals (BMI ≥ 24). Thus, the lncRNA *H19* gene rs2839698 polymorphism might be an important biomarker and diagnostic marker for predicting the susceptibility to CRC in Chinese Han population.

## 1 INTRODUCTION

Colorectal cancer (CRC) was among the most commonly diagnosed cancers and cancer‐related causes of death worldwide.^1^ The global number of CRC was supposed to increase by 60% to more than 2.2 million new patients and 1.1 million CRC‐related deaths by 2030.^2^ CRC was reported to be the world's 4th most deadly cancer with approximately 900 000 deaths annually.^3^ Studies showed that about 140 250 patients with CRC occurred in the United States in 2018.^4^ In China, CRC is the fourth and fifth most common cancer in women and men,^5^ respectively. Treatments for CRC included endoscopic and surgical local excision, chemotherapy and radiotherapy, targeted therapy, and immunotherapy and so on.^3^ The pathogenesis of CRC was complex and multifactorial, thus poorly understood. Although risk factors including smoking, excessive alcohol, excessive obesity, lack of physical exercise, and high consumption of red meat contributed to the risk of CRC,^3^, ^6^, ^7^ there were still CRC patients without exposure to these risk factors, indicating genetic susceptibility might play an important role in the pathogenesis of CRC.^2^ As reported, environmental factors, genetic factors, and their interactions contributed to the risk of CRC.^8^ Many studies have identified novel risk variants in patients with CRC.^9^, ^10^, ^11^, ^12^, ^13^, ^14^

Long non‐coding RNAs (lncRNAs), deemed as non‐protein‐coding transcripts longer than 200 nucleotides, were associated with crucial functions of various biological processes that affected cancer susceptibility.^15^, ^16^ LncRNAs played different roles in multiple physiological and pathological processes, including transcriptional regulation, posttranscriptional process, genome rearrangement, epigenetic control, chromatin modification, metabolic processes, and apoptosis.^17^, ^18^ Increasing evidence identified the key roles of lncRNAs in human diseases, especially in cancer.^19^, ^20^ The aberrant lncRNA expression may result in tumorigenesis.^21^ H19 was the first discovered lncRNA and was recognized as an important imprinted gene. LcnRNAH19 was related to cancer, and it was regarded as an important cancer biomarker for human cancer diagnosis and detection.^22^ Aberrant change in lncRNA H19 expression was shown in various cancers, suggesting a pivotal role of H19 in cancer progression.^23^ Cui et al^24^ indicated that H19 was upregulated in CRC. Yokoyama et al showed that changes in H19 were considered for predicting the susceptibility to 5‐FU‑based neoadjuvant chemotherapy in rectal cancer.^54^ H19 was also reported as a regulator of drug resistance in CRC.^25^, ^26^

The lncRNA *H19* gene is located on human chromosome 11p15.5, which contains four small introns and five exons.^27^ The single nucleotide polymorphisms (SNPs) in *H19* gene may affect its gene expression and function, thereby conferring the susceptibility to CRC. Several studies explored the association between the *H19* gene rs2839698 polymorphism and the risk of cancers,^18^, ^28^, ^29^, ^30^, ^31^, ^32^, ^33^ but the findings concerning different types of cancers were conflicting. Among these studies, Li et al^31^ found that *H19* gene rs2839698 polymorphism increased the risk of CRC. However, no other studies investigated the relationship between this SNP and CRC risk. Thus, we designed this case‐control study to assess the effects of *H19* gene rs2839698 polymorphism on the risk of CRC.

## 2 PATIENT AND METHODS

### 2.1 Subjects

This study enrolled 315 CRC cases and 441 gender‐ and age‐matched controls from the Wenzhou Hospital of Integrated Traditional Chinese and Western Medicine Affiliated to Zhejiang Chinese Medical University and the Second Affiliated Hospital of Zhejiang Chinese Medical University between 2012 and 2018. No patients with CRC received chemotherapy or radiotherapy before surgery. The histology of patients with CRC was histopathologically confirmed by two local pathologists. The family history, the tumor node metastasis (TNM) stage, histological grade, tumor size, location of CRC, lymph node metastasis, and the histology were collected from the medical records. The controls were selected from individuals receiving health examinations at the same period in these hospitals. The individuals with family history of cancer or digestive diseases, metastases from other origins, and history of radiotherapy or chemotherapy were excluded.

The demographic information of all subjects was retrieved through a structured questionnaire, including age, smoking status, gender, alcohol consumption, and family history of cancers. This study was approved by the Ethics Committees of the two hospitals and met the standards of *Declaration of Helsinki*. Written informed consent was obtained from each subject.

### 2.2 Blood sampling and genotyping

Peripheral blood (2 mL) was taken from each of the cases and controls. Genomic DNA was extracted from peripheral blood leukocytes by a TIANamp Blood DNA kit (Tiangen Biotech). The quality and concentration of the extracted DNA were measured at optical density (OD) wavelengths 260 and 280 nm using NanoDrop (Thermo Scientific). This SNP was genotyped using a fluorescent‐based restriction fragment length polymorphism method. The primers used for the nucleotide extension reaction were CATCGTCCCCAGCTGATGTC (forward) and GGAGTGATGACGGGTGGAG (reverse). PCR was performed in 25 μL of reaction mixture including 1.25 μL Genotyping Assays (20×), 20 ng DNA, 12.5 μL Genotyping Master Mix (2×). PCRs were carried out with an initial denaturation at 96°C for 5 minutes, followed by 35 cycles of 96°C for 30 seconds, annealing at 57°C for 40 seconds, and ending with a final elongation step at 72°C for 5 minutes. About 10% of the selected samples were randomly chosen for genotyping twice to ensure the genotyping accuracy,^34^, ^35^ and the results were 100% concordant.

### 2.3 Statistical analysis

The epidemiological variables of cases and controls were calculated using either Student's *t* test (continuous variables) or chi‐square (*χ* ^2^) test (categorical variables). The deviation between the observed and the expected frequencies among controls was evaluated by Hardy‐Weinberg equilibrium (HWE) test through a goodness‐of‐fit chi‐square test.^36^, ^37^ The relationship between the lncRNA *H19* gene rs2839698 polymorphism and the CRC risk was evaluated by computing the crude and adjusted odds ratios (ORs) and their 95% confidence intervals (CIs) using logistic regression analysis.^38^, ^39^ Subgroup analyses were conducted according to gender, age, alcohol, BMI, and smoking. In addition, the association between the lncRNA *H19* gene rs2839698 polymorphism and clinicopathologic features of patients with CRC was also investigated. All statistical analyses were analyzed on SPSS 22.0 (SPSS Inc) with the significance level at *P* < .05.

## 3 RESULT

### 3.1 Characteristics of the study population

Demographic information and clinical characteristics of the subjects are summarized in Table 1. The distributions of age, gender, smoking, and body mass index (BMI) did not differ significantly between the cases and the controls. The percentage of drinkers in the patients with CRC was significantly higher than in the controls (*P* < .001). Location of CRC showed that 113 cases were colon cancer and 202 were rectal cancer. The 315 patients with CRC included 300 cases with adenocarcinoma, 13 cases with squamous cell carcinoma, and 2 cases with other types of CRC. Based on the histological grade of cancer cell differentiation, 32, 248, and 35 CRC cases were well, moderately, and poorly differentiated, respectively. Other clinical characteristics of patients with CRC including lymph node metastasis, family history, TNM stage, tumor stage, and tumor size are shown in Table 1.

**TABLE 1:** Demographics and risk factors in colorectal cancer cases and controls

Characteristics	Case (N = 315)	Control (N = 441)	*P*
Age	63.35 ± 7.33	62.50 ± 7.33	.116
BMI	25.18 ± 1.45	25.15 ± 1.51	.802
Gender			.816
Male	60 (19.0%)	87 (19.7%)	
Female	255 (81.0%)	354 (80.3%)	
Smoking			**<.001**
Yes	204 (64.8%)	203 (46.0%)	
No	111 (35.2%)	238 (54.0%)	
Alcohol			**<.001**
Yes	277 (87.9%)	220 (49.9%)	
No	38 (12.1%)	221 (50.1%)	
Family history			
Yes	44 (14.0%)		
No	271 (86.0%)		
Histological grade			
Well differentiated	32 (10.2%)		
Moderately differentiated	248 (78.7%)		
Poorly differentiated	35 (11.1%)		
TNM stage			
I	67 (21.3%)		
II	90 (28.6%)		
III	93 (29.5%)		
IV	65 (20.6%)		
Tumor size			
>4 cm	165 (52.4%)		
≤4 cm	150 (47.6%)		
Lymph node metastasis			
No	182 (57.8%)		
Yes	133 (42.2%)		
Location of colorectal cancer			
Colon cancer	113 (35.9%)		
Rectal cancer	202 (64.1%)		
Pathology subtypes			
Adenocarcinoma	300 (95.1%)		
Squamous cell carcinoma	13 (4.1%)		
Others	2 (0.8%)		

Note

Bold values are significant results.

Abbreviations: BMI, body mass index; TNM, tumor node metastasis.

### 3.2 Relationship between lncRNA *H19* gene rs2839698 polymorphism and CRC risk

The genotype and allele distributions for the lncRNA *H19* gene rs2839698 polymorphism were significantly different between the patients with CRC and the controls (Table 2). The genotype frequencies of *H19* gene rs2839698 polymorphism were in accordance with HWE test among the control subjects. The AA genotype or AA + GA genotype carriers showed a significantly decreased risk for CRC (AA vs GG: OR, 0.73; 95% CI, 0.54‐0.98; *P* = .037; AA + GA vs GG: OR, 0.73; 95% CI, 0.54‐0.98; *P* = .037). Similarly, the A allele of the *H19* gene rs2839698 polymorphism was also associated with a decreased risk for CRC (A vs G: OR, 0.78; 95% CI, 0.63‐0.96; *P* = .021). However, the results were significant in dominant genetic models after adjusting for gender, age, BMI, alcohol, and smoking (AA + GA vs GG: adjusted OR, 0.69; 95% CI, 0.50‐0.96; *P* = .030).

**TABLE 2:** Association between H19 gene rs2839698 polymorphism and colorectal cancer risk determined by logistic regression analyses

Models	Genotype	Case (n, %)	Control (n, %)	OR (95% CI)	*P*‐value	OR (95% CI)*	*P*‐value*
Co‐dominant	GG	134 (42.7%)	154 (35.1%)	1.00	‐	1.00	‐
Heterozygote	GA	140 (44.6%)	211 (48.1%)	0.77 (0.56‐1.05)	.098	0.72 (0.50‐1.02)	.062
Homozygote	AA	40 (12.7%)	74 (16.9%)	**0.62 (0.40‐0.97)**	**.038**	0.63 (0.38‐1.02)	.062
Dominant	GG	134 (42.7%)	154 (35.1%)	1.00	‐	1.00	‐
	AA + GA	180 (57.3%)	285 (64.9%)	**0.73 (0.54‐0.98)**	**.037**	**0.69 (0.50‐0.96)**	**.030**
Recessive	GA + GG	274 (87.3%)	365 (83.1%)	1.00	‐	1.00	‐
	AA	40 (12.7%)	74 (16.9%)	0.72 (0.47‐1.09)	.118	0.75 (0.48‐1.18)	.214
Allele	G	408 (65.0%)	519 (59.1%)	1.00	‐	‐	‐
	A	220 (35.0%)	359 (40.9%)	**0.78 (0.63‐0.96)**	**.021**	**‐**	**‐**

Note

The genotyping was successful in 314 cases and 439 controls for rs2839698 polymorphism; Bold values are statistically significant (*P* < .05).

* Adjustment for age, sex, BMI, alcohol, and smoking.

Stratified analyses were analyzed according to gender, age, alcohol, smoking, and BMI (Table 3). A significantly decreased risk of CRC was found in non‐smokers (AA vs GG: OR, 0.49; 95% CI, 0.25‐0.93; *P* = .029), non‐drinkers, the subgroup of older patients with CRC (age ≥ 60 years), and overweight patients (BMI ≥ 24). However, stratified analysis by gender did not obtain positive findings. After adjusting for gender, age, alcohol, smoking, and BMI, similar results were obtained in Table S1. Those data indicated that *H19* gene rs2839698 polymorphism correlated with a decreased risk of CRC among non‐smokers, non‐drinkers, age ≥ 60 years subjects, and overweight individuals (BMI ≥ 24).

**TABLE 3:** Stratified analyses between H19 gene rs2839698 polymorphism and the risk of colorectal cancer

Variable	(case/control)			GA vs GG	AA vs GG	AA vs GG + GA	AA + GA vs GG
	GG	GA	AA				
Sex							
Male	30/33	24/44	6/10	0.60 (0.30‐1.21); 0.153	0.66 (0.21‐2.04); 0.470	0.86 (0.29‐2.50); 0.775	0.61 (0.31‐1.19); 0.147
Female	104/121	116/167	34/64	0.81 (0.57‐1.16); 0.251	0.62 (0.38‐1.01); 0.055	0.69 (0.44‐1.09); 0.112	0.76 (0.54‐1.05); 0.104
Smoking							
Yes	80/77	101/102	23/24	0.96 (0.63‐1.46); 0.858	0.92 (0.48‐1.77); 0.808	0.94 (0.51‐1.73); 0.849	0.96 (0.64‐1.42); 0.821
No	54/77	39/109	17/50	**0.51 (0.31‐0.85); 0.009**	**0.49 (0.25‐0.93); 0.029**	0.68 (0.37‐1.24); 0.211	**0.50 (0.32‐0.80); 0.004**
Alcohol							
Yes	109/83	131/97	36/40	1.04 (0.71‐1.53); 0.847	0.69 (0.40‐1.17); 0.165	0.67 (0.41‐1.10); 0.111	0.94 (0.65‐1.35); 0.718
No	25/71	9/114	4/34	**0.22 (0.10‐0.51); 0.001**	0.33 (0.11‐1.04); 0.058	0.64 (0.21‐1.92); 0.426	**0.25 (0.12‐0.52); 0.001**
Age (y)							
<60	28/56	36/68	14/23	1.06 (0.58‐1.94); 0.854	1.22 (0.55‐2.72); 0.632	1.18 (0.57‐2.45); 0.658	1.10 (0.62‐1.94); 0.746
≥60	106/98	104/143	26/51	**0.68 (0.47‐0.98); 0.041**	**0.47 (0.27‐0.81); 0.007**	**0.58 (0.35‐0.97); 0.037**	**0.62 (0.44‐0.89); 0.009**
BMI							
<24	28/38	25/43	11/11	0.79 (0.39‐1.58);0.503	1.36 (0.52‐3.57);0.536	1.53 (0.62‐3.78);0.356	0.91 (0.48‐1.73);0.761
≥24	106/116	115/168	29/63	0.75 (0.53‐1.07);0.110	**0.50 (0.30‐0.84);0.008**	**0.59 (0.37‐0.95);0.029**	**0.68 (0.49‐0.95);0.025**

Note

Bold values are statistically significant (*P* < .05).

### 3.3 Correlation between lncRNA *H19* gene rs2839698 polymorphism and clinicopathological characteristics of CRC patients

At last, the association between lncRNA *H19* gene rs2839698 polymorphism and the clinicopathologic features of patients with CRC was investigated (Table 4). The patients with CRC were not prone to lymph node metastasis (AA vs GG: OR, 0.43; 95% CI, 0.21‐0.88; *P* = .019). In addition, a significantly decreased risk was shown in CRC patients with tumor size < 4 cm (AA vs GG: OR, 0.42; 95% CI, 0.20‐0.88; *P* = .020). No association between this SNP and CRC risk was obtained regarding histological grade, TNM stage, family history, histology, and location of CRC. To sum up, lncRNA *H19* gene rs2839698 polymorphism was related with lymph node metastasis and the tumor size of CRC.

**TABLE 4:** The associations between H19 rs2839698 polymorphism and clinical characteristics of colorectal cancer

Characteristics	Genotype distributions			
	GG	GA	AA	GA + AA
Histological grade				
MD/WD	107/15	106/13	35/2	141/15
OR (95% CI); *P*‐value	1.0 (reference)	1.14 (0.52‐2.52); 0.740	2.45 (0.53‐11.26); 0.235	1.32 (0.62‐2.81); 0.475
Histological grade				
PD/WD	12/15	21/13	2/2	23/15
OR (95% CI); *P*‐value	1.0 (reference)	2.02 (0.72‐5.64); 0.178	1.25 (0.15‐10.23); 0.835	1.92 (0.71‐5.21); 0.200
TNM stage				
III + IV/I + II	66/68	70/70	22/18	92/88
OR (95% CI); *P*‐value	1.0 (reference)	1.03 (0.64‐1.66); 0.902	1.26 (0.62‐2.56); 0.524	1.08 (0.69‐1.69); 0.745
Tumor size				
≤4 cm/>4 cm	75/59	61/79	14/26	75/105
OR (95% CI); *P*‐value	1.0 (reference)	**0.61 (0.38‐0.98); 0.040**	**0.42 (0.20‐0.88); 0.020**	**0.56 (0.36‐0.88); 0.012**
Lymph node metastasis				
No/Yes	88/46	75/65	18/22	93/87
OR (95% CI); *P*‐value	1.0 (reference)	**0.60 (0.37‐0.98); 0.041**	**0.43 (0.21‐0.88); 0.019**	**0.56 (0.35‐0.89); 0.013**
Family history				
Yes/No	16/118	21/119	7/33	28/152
OR (95% CI); *P*‐value	1.0 (reference)	1.30 (0.65‐2.62); 0.459	1.56 (0.59‐4.12); 0.362	1.36 (0.70‐2.63); 0.361
Histology				
Adenocarcinoma/Not	126/8	135/5	38/2	173/7
OR (95% CI); *P*‐value	1.0 (reference)	1.71 (0.55‐5.38); 0.350	1.21 (0.25‐5.92); 0.817	1.24 (0.44‐3.53); 0.683
Location of colorectal cancer				
Colon cancer/Rectal cancer	48/86	47/93	18/22	65/115
OR (95% CI); *P*‐value	1.0 (reference)	0.91 (0.55‐1.49); 0.696	1.47 (0.72‐3.00); 0.294	0.75 (0.48‐1.19); 0.219

Note

Bold values are statistically significant (*P* < .05).

Abbreviations: MD, Moderately differentiation; PD, Poorly differentiation; WD, Well differentiation.

## 4 DISCUSSION

The *H19* gene rs2839698 polymorphism was shown to decrease the risk of CRC in a Chinese population. Stratified analyses showed *H19* gene rs2839698 polymorphism decreased the risk of CRC among non‐smokers, non‐drinkers, aged ≥ 60 years, and overweight individuals (BMI ≥ 24). In addition, the rs2839698 polymorphism was significantly correlated with lymph node metastasis and tumor size in patients with CRC.

LncRNAs are transcripts longer than 200 nucleotides, which could not be translated into proteins.^40^ LncRNAs were localized in the nucleus^41^ or cytoplasm.^42^ There were approximately more than 60 000 lncRNAs found in humans. LncRNAs could be further classified into four types: long intergenic ncRNAs (lincRNAs), pseudogenes, antisense RNAs (asRNAs), and circular RNAs (circRNAs). LncRNAs were the main components of the human transcriptome.^43^ LncRNAs were involved in biological processes by interfering with gene expression in some cancer types.^44^, ^45^ Recently, increasing studies revealed the critical roles of lncRNAs in the development of cancers.^19^, ^20^ The dysregulated lncRNAs regulated cell proliferation and apoptosis, invasion, epithelial‐to‐mesenchymal transition, migration, and drug resistance.^46^ In addition, some lncRNAs were biomarkers for the prognosis and diagnosis of some cancers.^40^ *H19*, a 2.3 kb intergenic and maternally expressed lncRNA, is located on chromosome 11p15.5. H19 had a pivotal role in cancer development including lung cancer, pancreatic cancer, ovarian cancer, bladder cancer, neuroblastoma, and CRC.^46^, ^47^, ^48^, ^49^ H19 was upregulated in CRC tissues when compared with adjacent noncancerous tissues.^50^, ^51^ H19 was associated with CRC survival and prognosis.^25^, ^52^ Furthermore, Qin et al^53^ indicated that *H19* gene polymorphisms might be functional biomarkers for predicting advanced CRC risk and prognosis.

Recently, several studies investigated the association between *H19* gene rs2839698 polymorphism and the risk of cancers. Verhaegh et al from the Netherlands showed that the TC genotype of rs2839698 polymorphism decreased the risk of bladder cancer, especially the developing non–muscle‐invasive bladder cancer.^28^ However, another study with 1049 bladder cancer cases and 1399 controls from China could not replicate the positive findings and revealed that rs2839698 polymorphism was not related to the risk of bladder cancer.^32^ Gong et al^30^ did not obtain any significant association between *H19* gene rs2839698 polymorphism and lung cancer susceptibility, but suggested this SNP was associated with a platinum‐based chemotherapy response in lung cancer. In addition, two studies showed *H19* gene rs2839698 polymorphism did not confer susceptibility to neuroblastoma in Chinese children.^18^, ^33^ Moreover, *H19* gene rs2839698 polymorphism increased the risk of gastric cancer in a Chinese Han population and the rs2839698 CT and TT genotypes were also associated with higher serum *H19* mRNA levels.^29^ Li et al^31^ observed that the A allele of rs2839698 polymorphism increased the risk of CRC and this SNP may change the crucial folding structures and the target microRNAs of *H19*. This present study showed *H19* gene rs2839698 polymorphism was associated with a deceased risk of CRC, which was different from the study by Li et al We thought the inconsistency may be attributed to four reasons. Firstly, our data indicated *H19* gene rs2839698 polymorphism interacted with some exposure factors, which were evidently diverse. Secondly, populations from different areas had different eating habits and living environments. Thirdly, differences in genotyping methods and the inclusion criteria may contribute to the inconsistent results. Fourthly, clinical heterogeneity of CRC may be attributed to the conflicting findings because the malignancy degree and pathological types of CRC differed among different studies.

The stratified analyses uncovered a decreased CRC risk in non‐smokers, non‐drinkers, aged ≥ 60 years, and overweight individuals (BMI ≥ 24). The above data suggested that the individuals exposed to these risk factors were not prone to CRC. Next, we explored the relationship between this SNP and clinicopathologic features of patients with CRC and uncovered that the CRC patients with rs2839698 polymorphism genotypes were not prone to lymph node metastasis and showed a decreased risk in the subgroup with tumor size < 4 cm. However, we did not obtain positive findings in the analyses of histological grade, TNM stage, family history, histology, or location of CRC. It should be noted that Li et al^31^ showed that colon tumor site, the well‐differentiated grade, and Duke's stage of C/D were significantly related to CRC risk. To sum up, our study indicated that lncRNA *H19* gene rs2839698 polymorphism was correlated with lymph node metastasis and the tumor size of CRC.

The advantages of this study included the following aspects: One, this is the first study to uncover the protective role of *H19* gene rs2839698 polymorphism in CRC development; two, we observed that *H19* gene rs2839698 polymorphism was related to a decreased risk for CRC in non‐smokers, non‐drinkers, and those aged ≥ 60 years; and three, the rs2839698 polymorphism was significantly correlated with lymph node metastasis and tumor size in patients with CRC. We think these abovementioned points were the innovations of this study, which were not found in other studies. However, this present study had several limitations. First, the sample size was not large, which may yield false‐positive results. Second, this case‐control study may have some weakness in identifying the cause‐effect relationship. Third, the lack of follow‐up data on patients with CRC limited further analysis. Fourth, the controls from the two hospitals may not fully represent the general population. Last, we only investigated one SNP of *H19* gene.

## 5 CONCLUSION

The lncRNA *H19* gene rs2839698 polymorphism is associated with deceased risk for CRC. Further studies with larger sample sizes are warranted to further verify this finding.

## CONFLICT OF INTEREST

The authors declare no competing interests.

## AUTHOR CONTRIBUTIONS

BingquYu, Jiayuan Chen, Chenfeng Hou, Lei Zhang, and Jie Jia conceived of the study and participated in its design. Bingqu Yu, Jiayuan Chen, Chenfeng Hou, Lei Zhang, and Jie Jia conducted the systematic literature review. Jie Jia performed data analyses. Bingqu Yu, Jiayuan Chen, Chenfeng Hou, Lei Zhang, and Jie Jia drafted the study. All gave final approval and agree to be accountable for all aspects of work ensuring integrity and accuracy.

## Supporting information

Table S1
