# Supplementary material for: LncRNA H19 gene rs2839698 polymorphism is associated with a decreased risk of colorectal cancer in a Chinese Han population: A case‐control study
Source: J Clin Lab Anal. 2020 Mar 24;34(8):e23311. doi: 10.1002/jcla.23311 (PMC7439357; doi:10.1002/jcla.23311)
Supplement: Supplementary file 1 — Table S1 [file JCLA-34-e23311-s001.doc]

**Supplemental Table 1** Stratified analyses between H19 gene rs2839698 polymorphism and the risk of colorectal cancer.

| Variable | rs2839698 (case/control) | | | GA vs. GG | AA vs. GG | AA vs. GG+GA | AA+GA vs. GG |
| --- | --- | --- | --- | --- | --- | --- | --- |
|  | GG | GA | AA | ^a^OR (95% CI), ^a^*P*-value | ^a^OR (95% CI), ^a^*P*-value | ^a^OR (95% CI), ^a^*P*-value | ^a^OR (95% CI), ^a^*P*-value |
| Sex |  |  |  |  |  |  |  |
| Male | 30/33 | 24/44 | 6/10 | 0.75(0.35-1.63); 0.467 | 0.77(0.23-2.65); 0.682 | 0.82(0.26-2.60); 0.736 | 0.75(0.36-1.57); 0.448 |
| Female | 104/121 | 116/167 | 34/64 | 0.72(0.48-1.07); 0.106 | 0.63(0.37-1.06); 0.081 | 0.74(0.45-1.21); 0.232 | 0.69(0.47-1.01); 0.051 |
| Smoking |  |  |  |  |  |  |  |
| Yes | 80/77 | 101/102 | 23/24 | 0.88(0.57-1.38); 0.843 | 0.74(0.37-1.57); 0.637 | 0.92(0.57-1.63); 0.743 | 0.88(0.57-1.33); 0.933 |
| No | 54/77 | 39/109 | 17/50 | **0.43(0.25-0.76); 0.004** | **0.37(0.18-0.76); 0.006** | 0.54(0.28-1.16); 0.223 | **0.43(0.26-0.72); 0.001** |
| Alcohol |  |  |  |  |  |  |  |
| Yes | 109/83 | 131/97 | 36/40 | 1.13(0.82-1.43); 0.946 | 0.73(0.41-1.19); 0.196 | 0.63(0.38-1.21); 0.133 | 0.82(0.75-1.44); 0.732 |
| No | 25/71 | 9/114 | 4/34 | **0.23(0.10-0.52); 0.001** | 0.38(0.23-1.14); 0.067 | 0.63(0.24-1.93); 0.344 | **0.28(0.13-0.57); 0.001** |
| Age (years) |  |  |  |  |  |  |  |
| <60 | 28/56 | 36/68 | 14/23 | 1.13(0.63-2.04); 0.832 | 1.13(0.45-2.68); 0.335 | 1.21(0.32-2.31); 0.548 | 1.22(0.57-1.84); 0.866 |
| ≥60 | 106/98 | 104/143 | 26/51 | **0.63(0.42-0.96); 0.032** | **0.53(0.30-0.95); 0.034** | 0.67(0.38-1.17); 0.161 | **0.60(0.41-0.90); 0.012** |
| BMI |  |  |  |  |  |  |  |
| <24 | 28/38 | 25/43 | 11/11 | 0.83(0.43-1.32);0.612 | 1.45(0.63-3.69);0.546 | 1.48(0.73-3.24);0.436 | 0.78(0.33-1.64);0.778 |
| ≥24 | 106/116 | 115/168 | 29/63 | 0.64(0.63-1.22);0.133 | **0.54(0.31-0.94);0.028** | 0.63(0.38-1.07);0.089 | **0.65(0.44-0.95);0.027** |

^a^Adjustment for sex, smoking, alcohol, gender and BMI, omitting the corresponding stratification factor. Bold values are statistically significant (*P* <0.05).
